# Optimizing Gestational Weight Gain With the Eating4Two Smartphone App: Protocol for a Randomized Controlled Trial

**DOI:** 10.2196/resprot.9920

**Published:** 2018-05-30

**Authors:** Deborah Davis, Rachel Davey, Lauren T Williams, Maralyn Foureur, Ellen Nohr, Catherine Knight-Agarwal, Tanya Lawlis, Jeremy Oats, Helen Skouteris, Matthew Fuller-Tyszkiewicz

**Affiliations:** ^1^ University of Canberra Canberra Australia; ^2^ ACT Government Health Directorate Canberra Australia; ^3^ Centre for Research & Action in Public Health, Health Research Institute University of Canberra Canberra Australia; ^4^ Menzies Health Institute of Queensland Griffith University Southport Australia; ^5^ Centre for Midwifery, Child and Family Health University of Technology Sydney Sydney Australia; ^6^ Institute of Clinical Research University of Southern Denmark Odense Australia; ^7^ Faculty of Health University of Canberra Sydney Australia; ^8^ Melbourne School Population & Global Health The University of Melbourne Melbourne Australia; ^9^ Monash Centre for Health Research and Implementation School of Public Health and Preventive Medicine Monash University Clayton Australia; ^10^ Deakin University Waurn Ponds Australia

**Keywords:** smartphone, technology, prenatal care, pregnancy, weight gain

## Abstract

**Background:**

Approximately 50% of women gain excessive weight in pregnancy. Optimizing gestational weight gain is important for the short- and long-term health of the childbearing woman and her baby. Despite this, there is no recommendation for routine weighing in pregnancy, and weight is a topic that many maternity care providers avoid. Resource-intensive interventions have mainly targeted overweight and obese women with variable results. Few studies have examined the way that socioeconomic status might influence the effectiveness or acceptability of an intervention to participants. Given the scale of the problem of maternal weight gain, maternity services will be unlikely to sustain resource intensive interventions; therefore, innovative strategies are required to assist women to manage weight gain in pregnancy.

**Objective:**

The primary aim of the trial was to examine the effectiveness of the Eating4Two smartphone app in assisting women of all body mass index categories to optimize gestational weight gain. Secondary aims include comparing childbirth outcomes and satisfaction with antenatal care and examining the way that relative advantage and disadvantage might influence engagement with and acceptability of the intervention.

**Methods:**

This randomized controlled trial will randomize 1330 women to control or intervention groups in 3 regions of different socioeconomic status. Women will be recruited from clinical and social media sites. The intervention group will be provided with access to the Eating4Two mobile phone app which provides nutrition and dietary information specifically tailored for pregnancy, advice on food serving sizes, and a graph that illustrates women’s weight change in relation to the range recommended by the Institute of Medicine. Women will be encouraged to use the app to prompt conversations with their maternity care providers about weight gain in pregnancy. The control group will receive routine antenatal care.

**Results:**

Recruitment has commenced though the recruitment rate is slower than expected. Additional funds are required to employ research assistants and promote the study in an advertising campaign.

**Conclusion:**

Feasibility testing highlighted the inadequacy of the original recruitment strategy and the need to provide the app in both major platforms (Android and iOS). Smartphone technologies may offer an effective alternative to resource intensive strategies for assisting women to optimize weight gain in pregnancy.

**Trial Registration:**

Australian New Zealand Clinical Trials Registry ACTRN12617000169347; https://www.anzctr.org.au/Trial/Registration/TrialReview.aspx?id=371470 (Archived by WebCite at http://www.webcitation.org /6zDvgw5bo)

**Registered Report Identifier:**

RR1-10.2196/9920

## Introduction

### Background

Obesity is one of the most significant health issues of our time. Regardless of prepregnancy body mass index (BMI), the amount of weight gained during pregnancy (gestational weight gain, GWG) has the potential to impact the health and well-being of the childbearing woman and her baby in the short, medium, and long term. Women who gain excessive weight in pregnancy are more likely to retain weight in the short, medium and long term, progressing from normal weight to obese over their childbearing years. There is a critical need to reduce the burden of maternal obesity and excessive GWG with programs that are effective, accessible, and scalable for delivery at a population level, and to understand how socioeconomic factors impact GWG and uptake of interventions. This research proposal addresses these 2 key issues by examining the efficacy of the Eating4Two smartphone app in a population of pregnant women of all BMI categories and varying levels of socioeconomic advantage and disadvantage.

Optimizing GWG is important to the short- and long-term health of the childbearing woman and her baby. Excess GWG is associated with maternal hypertensive disease, and caesarean section [1] is an independent risk factor for large for gestational age infants [2], and may have long-term consequences for the neonate. Independent of maternal BMI, excessive GWG is associated with childhood adiposity at birth [3] and 3 [4] and 5 years of age [5]. These data suggest that careful management of gestational weight gain is as important for the offspring of underweight and normal weight women as it is for the overweight and obese.

Excessive gestational weight gain may also influence maternal health beyond the duration of the pregnancy. A systematic review and meta-analysis of studies examining maternal weight gain, neonatal outcomes, and maternal weight retention found that women who experienced excess GWG were more likely to retain excess weight in the short, medium, and long term [6]. This has implications for subsequent pregnancy outcomes. Our previous work has shown that multiparous women who had an interpregnancy increase of 3 BMI units or more had significantly increased odds of low 5-min Apgar score, gestational diabetes, and hypertensive disorders in the subsequent pregnancy in adjusted analyses, independent of previous BMI [7]. There are also consequences for long-term chronic disease risk [8]. Maternal and neonatal outcomes are optimized when women begin their pregnancy at a healthy weight and maintain a healthy weight throughout pregnancy. Maternal overweight and obesity is associated with increased risks of gestational diabetes, hypertension, induction of labor, stillbirth, preterm birth, major congenital malformations, large babies, shoulder dystocia, caesarean section, wound infections, and postpartum hemorrhage [9-13]. These risks increase with increasing maternal BMI [10]. Prepregnancy maternal weight also has long-term consequences for the neonate. Babies born to women who are overweight or obese have a higher likelihood of childhood overweight and obesity [14]. Although the mechanism underpinning this intergenerational effect is not yet clear, some researchers (though not consistently) have identified epigenetic modifications in offspring of under- and over-nourished women [15]. Intergenerational patterns of obesity and the potential for epigenetic effects in the offspring of overweight and obese women, means that childbearing women are a critical target population for interventions aimed at addressing obesity in the general population.

Table 1 shows the gestational weight gain recommendations of the Institute of Medicine (IOM) for women of varying BMI categories [16]. Although these recommendations are officially endorsed in Australia by the Royal Australian and New Zealand College of Obstetricians and Gynecologists, the recommendations for weight gain in pregnancy they are rarely referred to in practice. The prevalence of GWG in excess of the IOM guidelines in developed countries is approximately 50% [16]. Of concern is that younger and nulliparous women have a greater likelihood of gaining excess weight during pregnancy. Pregnancy is a significant life event for women and it is a time that many women are motivated to focus on their health. Furthermore, pregnancy offers an opportunity to positively impact the short- and longer-term health of 2 individuals with a single intervention.

**Table 1:** Institute of Medicine gestational weight gain recommendations.

Prepregnancy BMI^a^ (kg/m^2^)	Recommended weight gain (kg)
<18.5	12.5-18
18.5-24.9	11.5-16
25-29.9	7-11.5
>30	5-9

^a^BMI: body mass index.

Various resource-intensive interventions have been developed to assist overweight and obese women to manage their weight gain in pregnancy with varied success. These include one-to-one dietary counseling and targeted exercise programs [17]. The LIMIT trial, for example [18], randomized over 2000 overweight and obese women to a control condition or antenatal dietary and lifestyle intervention and found no differences in maternal pregnancy and birth outcomes, GWG or risk of large-for-gestational-age babies between groups. Babies born to mothers in the intervention group, however, were significantly less likely to have a birth weight greater than 4000 g.

The UPBEAT trial in the United Kingdom [19] randomized 1555 obese women to control or to an intervention that included coaching by a health trainer at enrolment and a further 8 individual or group sessions over the pregnancy. This study identified no differences between control and intervention groups in the primary outcomes of gestational diabetes and large-for-gestational-age infants; however, there was a small but significant difference in total GWG with women in the intervention group gaining 0.55 kg less than those in the control group. A recent systematic review with economic analysis of diet and lifestyle interventions aiming to assist with GWG found no evidence that interventions in pregnancy are cost effective or clinically effective [20]. Given the magnitude of the problem of maternal weight gain in the population and the already stretched resources of maternity services, it is unlikely that such resource-intensive interventions will be sustainable even if they do prove to be effective. New and innovative ways of addressing GWG in a sustainable way that can benefit all pregnant women, not just those who are overweight or obese, are a high priority.

### Development of an App to Optimize Gestational Weight Gain

**Figure 1:**
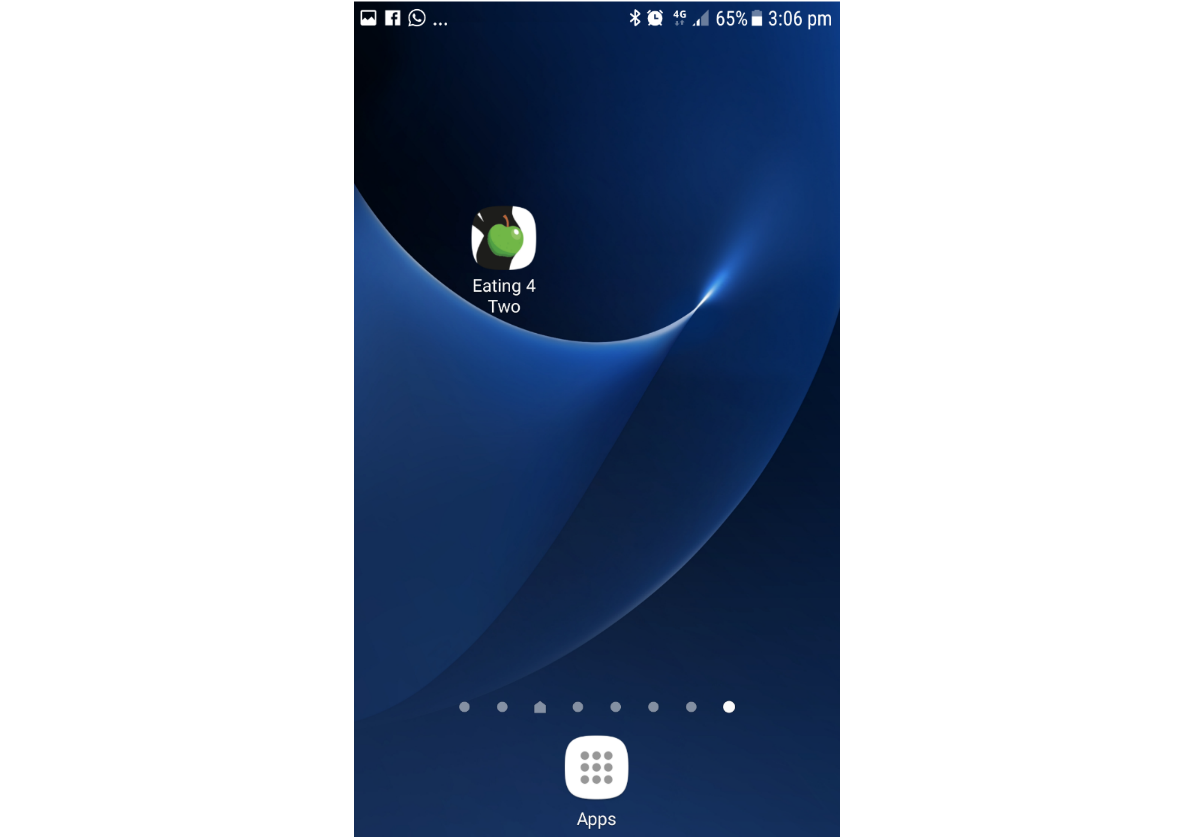
Eating4Two App icon.

**Figure 2:**
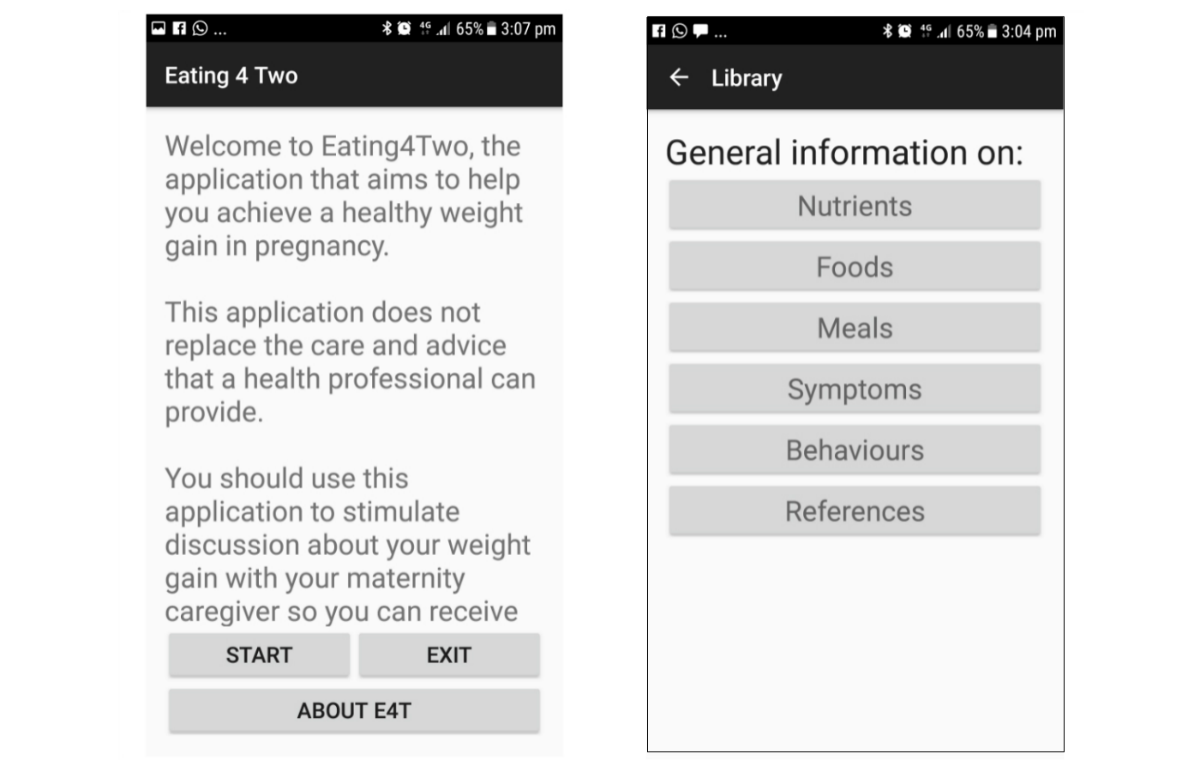
Eating4Two welcome screen (left) and library screenshot (right).

This led us to the development of a mobile phone app: Eating4Two (the app) [21] (see Figure 1 for app icon). Experts in nutrition and dietetics, public health, midwifery, and obstetrics contributed to its development drawing on the information-motivation-behavioral skills approach to behavior change [22]. Childbearing women were involved in various stages of the development. The welcome screen reminds users that the aim of the app is to augment rather than replace maternity care (see Figure 2 [left] for welcome screen).

**Figure 3:**
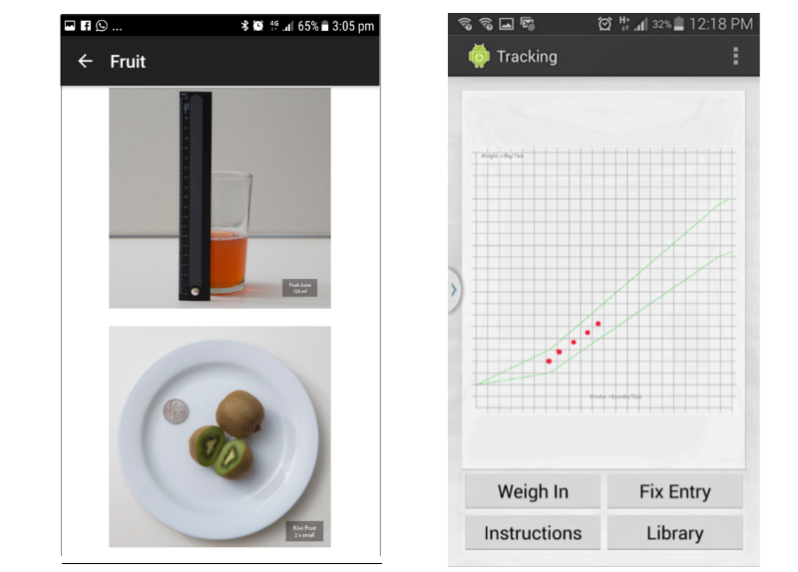
Eating4Two recommended serving size fruit (example; left) and weight tracker (right).

The app (developed originally in the Android platform and now available in Android and iOS) is aimed at all pregnant women (in every BMI range), providing them with information on diet and nutrition in pregnancy drawing on the National Health and Medical Research Council’s Nutrient Reference Values for Australia and New Zealand [23]. The app contains a library of information (see Figure 2, right) that includes the following tabs: nutrients (describing important nutrients required for pregnancy and foods that contain them), foods (describing the main food groups and recommended serving sizes; see, eg, Figure 3, left), meals, (providing sample meal plans), symptoms (advice on how to manage common pregnancy symptoms, eg, heartburn), behaviors (providing advice on lifestyle behaviors including use of alcohol and managing cravings), and references.

BMI is calculated based on self-reported prepregnancy weight and height, and based on this information, a tailored gestational weight gain range is calculated. Women are encouraged to plot their weight on a graph weekly (which provides real time feedback on their GWG) based on the IOM recommendations (see Figure 3 [right] for weight tracker).

Self-monitoring of weight has been identified as an effective strategy in weight loss interventions [24]. Women are provided with instruction on how to weigh themselves and are asked to focus on trends rather than individual results. When weight gain is above or below the recommended range, women are prompted to discuss the issue with their maternity caregiver so that individualized advice can be provided. The app is intended to augment maternity care and to empower pregnant women to manage their GWG. In addition, push notifications are delivered based on the individual’s gestation, providing information on the size of the fetus and relevant nutritional information. No prompts or reminders are used to encourage use of the app.

Mobile phones have the potential for scalability of interventions, given their ubiquitous distribution in Australia. The Australian Bureau of Statistics reports that as of June 2017, there were 26.3 million mobile phone subscribers with access to the Internet in Australia [25]. Smartphone ownership in the 25-34 years and 35-44 years age groups (the main childbearing years) in Australia is 85% and 81%, respectively. In the 6 months leading up to May 2013, 76% and 66% of people in these age ranges had downloaded a mobile phone app [26]. A small qualitative study focusing on pregnant women in South Australia (*n*=35) found that all participants had access to a mobile phone and that the majority of these were smartphones [27]. Moreover, 40% of these participants reported having used at least one pregnancy-related mobile phone app.

Despite the rapid increase in the number of apps and other mHealth interventions available in the field of maternal and newborn health [28], there is a paucity of robust studies that objectively measure clinical outcomes for the mother or newborn [29-31]. Much of the research has focused on middle- and low-income countries [30,31] due to the high need for interventions to improve poor perinatal outcomes in these low-resource settings and rapidly improving mobile technology infrastructure. Studies have shown that mHealth interventions (short message service, SMS, support) has improved service utilization and vaccinations rates [29], reduced risk of perinatal death, and improved breastfeeding rates [30]. Focusing more globally, Chen’s recent [28] systematic review highlights the rapid increase in studies focusing on mHealth interventions, noting that interventions in resource-poor countries tended to focus on infections disease and essential care, whereas those from developed countries focused on noncommunicable diseases such as asthma or diabetes.

Despite the increase in studies, Chen et al concur with others when they comment on the poor quality of many studies in this area.

Mobile phone apps are developed by individuals, groups, and organizations representing public and private interests, and vary greatly in their quality. Health care professionals are also increasingly turning to mobile phone apps to deliver health care interventions, particularly in the area of preventative health [32]. A systematic review examining new communication technologies and their potential to support lifestyle interventions during pregnancy [33] identified 7 completed and ongoing studies using a range of technologies from websites (3), video (1), phone (1), and smartphone apps (2). The latter group included one pilot study focusing on physical activity and pregnancy [34] and one ongoing study focusing on GWG in the United States [35]. Targeting overweight and obese women, this 3-arm randomized control trial includes the following: standard care (physician directed), regular meetings with a weight management counselor during pregnancy (SmartMoms-clinic), and 2 individual meetings with a weight management counselor, followed by weekly messages via smartphone from the counselor (SmartMoms-phone).

Western Australian researchers have developed a Web-based resource, *Healthy You, Healthy Baby* [36]. The website and app are aimed at providing women with information on a range of topics including nutrition, physical activity, weight, emotions, social life, and sleep patterns. The researchers estimate that the website and app have been accessed by approximately 7% of all pregnant women in Western Australia and report that the section on weight was the most highly accessed. Although the effectiveness of this intervention is yet to be established by a research trial, the authors conclude that the strategy is a cost-effective way of providing women with accurate information about weight and other relevant perinatal issues [36].

Interventions that target individual behavior (rather than structural change) have been criticized for increasing the socioeconomic gradient of obesity [37]. The burden of overweight and obesity is felt most significantly in populations from lower *socioeconomic positions*. As those with fewer resources are less able to make the changes required of behavioral interventions, these types of interventions are hypothesized to impact most positively on those who can (those in the highest socioeconomic position). There is some support for this hypothesis coming from a study on an Internet-based intervention for limiting GWG in the United States [38]. The researchers measured engagement with the Web-based interventions according to demographic variables. Although they were generally encouraged by the engagement of all groups with the intervention, they found that white, higher income women were more consistently engaged with the Web-based resource than minority and low-income women. Again, the weight tracker was the most highly accessed resource within the intervention.

Despite the ubiquity of mobile phones in Australia and the growing use of this technology by researchers in health care more broadly, there is a paucity of research testing interventions with this technology in pregnancy. Intensive interventions for GWG, and overweight and obesity in maternity, are beyond the capacity of existing services. New and innovation solutions are required. The intervention developed is an innovative and potentially scalable solution that aims to empower women to take control of the issue in partnership with their maternity care provider. Importantly, the study will also examine the implementation of the intervention in areas with a different Index of Relative Socioeconomic Advantage and Disadvantage (IRSAD).

### Study Focus and Aims

Using a randomized control design with qualitative components, the RCT study will primarily examine the effectiveness of the Eating4Two smartphone app in assisting pregnant women to limit GWG to within the range recommended by the IOM. In addition, it will examine the acceptability of the intervention and engagement with the app for women with varying indices of relative socioeconomic advantage and disadvantage (IRSAD). The secondary aims of the study include examining clinical outcomes and satisfaction with perinatal care for women in both groups and drawing on qualitative data, comparing the experiences and challenges of women from areas with varying IRSAD.

## Methods

### Study Design and Overview

This study is an unblinded, 2-armed randomized control trial that will test the primary hypothesis that a greater proportion of women in the intervention group compared with the control group will limit GWG within the range recommended by the IOM. We will randomize 1330 pregnant women to the control and intervention groups. Those in the control group will receive usual antenatal care, and those in the intervention group will receive usual care augmented with the smartphone app. Participants allocated to the intervention group are prompted by the smartphone app to discuss weight changes with their maternity or health care provider. The researcher responsible for analyzing the data will be blind to group allocation.

### Settings

The proposed study will be implemented in 3 geographic regions. Region 1 is the Australian Capital Territory (ACT) and will include women attending the Centenary Hospital for Women and Children (a tertiary-level hospital) and Calvary Hospital (a sub-acute facility). These hospitals facilitate over 5000 births per annum [39]. Region 2 is the Hunter New England region of NSW, Australia, and will include women attending the John Hunter (a tertiary-level hospital) and Maitland (a sub-acute facility) hospitals. These hospitals facilitate approximately 5700 births per annum [40]. Region 3 is Port Macquarie-Hastings and includes women attending Port Macquarie Base Hospital (a sub-acute facility) that facilitates approximately 2000 births per annum.

The index of relative socioeconomic advantage and disadvantage, developed by the Australian Bureau of Statistics draws on census data to describe the economic and social conditions of people and households in a geographical area, the smallest area being a Statistical Area 1 (SA1), which includes approximately 400 people. A low score reflects greater disadvantage and a high score greater advantage [41]. The ACT as a whole, has an IRSAD decile ranking of 10 though individual suburbs range in decile rankings from 7-10. The Newcastle local government area (in which the John Hunter and Maitland Hospitals are situated) has a ranking of 7 and draws on areas with rankings that range from 3 to 7; Port Macquarie has a ranking of 5, drawing on areas with rankings ranging from 1 to 10.

In all settings, private obstetricians provide maternity care with antenatal appointments occurring in their private rooms. Their clientele will give birth in either in public or private hospitals. Public maternity care is provided by many health practitioners (including midwives, general practitioners, obstetricians or obstetricians in training) in community and hospital settings in one of the 2 models. First, women choosing a *shared care* model will have some antenatal appointments with general practitioners in their clinic rooms and some in the hospital-based antenatal clinic where they will be seen by employed obstetricians, obstetricians in training or midwives. Second, women choosing a public *midwifery led* model of care will primarily see midwives throughout the antenatal period in the antenatal clinic, birth center, and sometimes community settings. Both groups will be eligible to participate in the study. Labor and birth care is provided in the birth units of the participating hospitals.

### Participants

Participants will be pregnant women who plan to give birth in 1 of the 5 participating hospitals. Inclusion criteria for participants are as follows: (1) 18 years of age or older, (2) ability to provide informed consent, (3) fluent in written and oral English language, (4) less than 15 weeks gestation at recruitment, (5) personal ownership of a smartphone, (6) access to weighing scales, and (7) a valid email address and access to Internet. Potential participants will be excluded if they are planning to give birth in a nonparticipating hospital; have a multiple pregnancy, Type 1 or 2 diabetes before pregnancy, and barriers to accessing or using a smartphone for the duration of the trial; or their health care provider considers use of the app or GWG in accordance with IOM recommendations detrimental to the potential participant.

### Recruitment and Randomization

Recruitment will be via social media and health professionals providing antenatal care or childbirth education. Social media will publish the research aims and eligibility criteria and invite potential participants to contact the research assistant who will provide more information and a research information pack. Health professionals will introduce the study to potential participants and provide those interested and eligible with a research information pack. They will also collect contact details of those expressing an interest (for follow up by the research assistant). The research information pack contains a concise lay description of the study, contact details of the researchers, a hard copy questionnaire to establish baseline demographic details, consent form, and reply-paid envelope. The research assistant will contact all those provided with an information pack to discuss potential participation and answer any questions they may have. Those who agree to participate will complete the consent form and questionnaire and return these to the research coordinator in the reply-paid envelope provided. The research coordinator will record the date of receipt.

The research coordinator will randomize participants on receipt of the signed consent form. Participants will be randomized using a 1:1 ratio in balanced blocks and stratified by BMI and geographical setting. Allocation concealment will be assured through the use of a remote Web-based allocation service.

### Control and Intervention

The control group will receive usual antenatal maternity care with the addition of a written nutrition and weight gain resource: the booklet “Good nutrition in pregnancy,” published by the ACT Government. Usual care for women includes attendance at antenatal appointments in accordance with the following gestation schedule: 12-14 weeks (booking visit), 16, 20, 26, 30, 33, 36, 38, 40, and 41 weeks. Women choosing shared care usually alternate visits with their general practitioner and hospital clinic. Although women’s BMI is calculated at their first hospital-booking visit, weighing is not routinely attended at subsequent antenatal clinic appointments or at labor commencement. Advice on weight gain is ad hoc, and dietary advice is focused on food safety in pregnancy. Women with a BMI over 35 kg/m^2^ are referred to a community dietitian. Some amendment to usual care will be required to weigh women at 38 weeks and at commencement of labor.

The intervention group will receive usual care as outlined above and will also be provided access to the Eating4Two smartphone app at no cost. The app, password protected, will be able to be downloaded from App stores. The app provides dietary information appropriate to pregnancy and advice on good nutrition (drawn from the Australian Dietary Guidelines 2017 [23]), managing common pregnancy-related symptoms (such as heart burn), and GWG. Photographs are included that demonstrate serving sizes for relevant food groups. The woman’s weight is graphed against the range recommended by the IOM for their starting BMI and records this information, making it available for the researchers. When weight gain is above or below the recommended range, women are prompted to discuss the issue with their maternity caregiver so that individual advice can be provided. The app also sends regular messages to the participant (specific to their gestation) with information about their baby’s growth, development and nutritional needs, motivational messages, tips on weight management and physical activity in pregnancy, and reminders to discuss weight gain with their maternity caregivers. The aim of the app is to augment usual antenatal care by providing information to women about nutrition, empowering them to monitor their GWG, and encouraging discussion of weight between women and their maternity caregivers when GWG deviates from the recommended levels.

### Data Collection

The weight of all women will be established prepregnancy (self-reported), pregnancy (at enrolment in study <15 weeks gestation, self-reported), at approximately 38 weeks gestation (measured), commencement of labor (measured), and at 6 months postpartum (self-reported). The weight of women in the intervention group as recorded by the women in the app will also be available to researchers. Routinely collected clinical maternity data will be used where possible and this will be drawn from clinical records. This includes demographic data (age, parity, ethnicity and address) and behaviors including smoking and clinical outcomes. Using geo-coding, addresses of women participating in this study will be matched to the Australian Bureau of Statistics geographical data and allocated an IRSAD score. Data not collected routinely (including education and marital status) will be sought from participants (via a questionnaire), which will include participation in exercise and dietary intake established at baseline and repeated at approximately 38 weeks gestation, information about the GWG resources and advice accessed by women (38 weeks) and their rating of the app (38 weeks for intervention group only). Satisfaction with perinatal care will be assessed by questionnaire at 4-6 weeks postpartum.

The primary outcome will be GWG, specifically the proportion of women whose GWG falls within the range recommended for them by the IOM for their baseline BMI. GWG will be calculated by subtracting the woman’s first recorded pregnancy weight from her recorded weight in labor. Secondary outcomes including pregnancy complications, labor interventions, mode of birth, and neonatal outcomes will be drawn from routinely collected maternity outcome data and will include the following data: complications arising in pregnancy including hypertension and gestational diabetes (as diagnosed by health professional), labor interventions (including induction or augmentation of labor), mode of birth (spontaneous vaginal, assisted vaginal, caesarean section), birth complications (eg, shoulder dystocia), and neonatal outcomes (including gestation, birth weight, admission to neonatal nursery, Apgar score).

Women allocated to the intervention group will be required to enter the following initial data into the Eating4Two app: height, prepregnancy weight, and baby’s due date. Thereafter, they will be requested to enter their weight on a weekly basis. Data transferred to the researchers directly from the app include height, prepregnancy weight, baby’s due date, and weekly weights. Four to six weeks after the participant’s due date, they will be sent an email containing a link to a Web-based questionnaire in Qualtrics assessing quality of perinatal care. The Quality Perinatal Care Questionnaire is a 46-item instrument with 6 validated subscales originally developed in Canada and validated in an Australian population [42]. This email will also contain an invitation to participate in a focus group or individual interview. This component seeks to understand the barriers and facilitators to optimal GWG in pregnancy from a personal, social, and health care perspective, and specifically to understand how these may differ for women of different prepregnancy BMI and IRSAD (index of relative social advantage and disadvantage) groups. Those in the intervention group will also be asked specific questions about the app, which will provide additional information on the components of the App and its acceptability, functionality, engagement, and motivational capacity.

Additionally, this seeks to explore how BMI and IRSAD might impact app evaluation. Focus groups will aim for between 8 to 12 participants in each session, and intervention and control group women will attend separate groups. We will also attempt to group women living in areas with similar IRSAD scores and prepregnancy BMI categories together so that we can examine the ways in which these factors might impact issues relating to gestational weight management, engagement with maternity care providers, and engagement with the Eating4Two app(for those in intervention group). We will aim for 8 focus groups in total (64-96 participants). Individual interviews (by phone) will be offered to those who cannot attend focus groups.

It can be difficult for new mothers to attend scheduled focus groups, and individual phone interviews offer greater flexibility. We will aim for a total of 20 individual interviews balanced between intervention and control groups and sites. All focus groups and interviews will be audio recorded and transcribed verbatim.

### Data Analysis

Primary analysis will be conducted with the researcher responsible for analysis blinded to group allocation. An intention-to-treat analysis will be conducted including withdrawals and losses to follow-up. Although even distribution of baseline characteristics between the intervention and the control groups is expected due to randomization, this will be further assessed by Chi-squared test and Student *t*-test for categorical and continuous variables, respectively. Descriptive statistics for all outcome variables will be calculated before statistical analysis. Continuous outcome variables will be evaluated for normality and transformations will be applied as necessary. Regression analysis with adjustments for confounding variables (prepregnancy BMI, smoking, IRSAD score, parity, age) will be used to evaluate the primary outcome and relative risk, and its 95% CIs will be calculated. Significance will be set at .05. The multiple imputation method will be used to generate possible values for missing values. This is considered gold standard for dealing with missing data. Data will be analyzed in IBM SPSS Statistics).

Qualitative data analysis will firstly follow a simple descriptive approach using NVIVO 11 software (QRS International). Qualitative descriptive analysis is a low-inference analysis that uses an inductive approach to develop descriptive themes. All transcripts will be coded by first attaching a descriptive label to each meaning unit (a sentence or group of sentences conveying a message or concept relevant to the study); first level. Descriptive labels will be examined and grouped with other labels conveying a similar idea to create descriptive themes; second level. Data will be analyzed within groupings (BMI and IRSAD) and then compared across groups; third level. This will be attended by 2 researchers independently (first level) and then collaboratively on the second and third levels of analyses.

### Sample Size

This trial is designed to detect a clinically significant increase in the proportion of women who have a gestational weight gain within the current IOM recommendations from 36% to 42%. A recent prospective study in Australia with 664 participants (of all BMI categories) found that 36% of the cohort gained within the range recommended by the IOM [43]. This is greater than the proportion found in the LIMIT trial (33%), though this trial included only overweight and obese women [18] who are known to be more at risk of excessive weight gain. A total sample size of 1156 (578 in each group) will allow detection of statistical significance with 80% power and two-sided 5% significance level [44].

The follow-up period is short in studies using GWG as the primary outcome, given the end point is birth. However, there is wide variation in reported drop-out and loss to follow-up rates, which range from 3% to 20% [18,45-47]. These are composed largely of women experiencing miscarriage, fetal loss, and moving out of area. Taking a conservative approach, we have allowed for a 15% drop-out rate, which gives a total recruitment target of 1330 women.

### Ethics

The study received multi-site ethics approval from the ACT Health Human Research Ethics Committee (ETH.5.16.064). All potential participants will be supported to make an informed choice regarding joining the study and will be required to sign a consent form before enrolment. Consent includes consent to access the individual’s data from clinical databases at each participating hospital. Individual data will not be reported and participant confidentiality will be protected.

### Trial Status

Due to resource limitations, the Eating4Two mobile phone app was originally developed in the Android platform only. Over 4 months from October 2014 to February 2015, we attempted to recruit 80 women at one site only to determine study feasibility. Eligibility for the study included ownership of an android smartphone. Exclusion criteria included gestation greater than 18 weeks, multiple pregnancy, and preexisting clinical conditions including diabetes. A research assistant approached women waiting for antenatal appointments at the busiest antenatal clinics in the hospital and in the community, 2 days per week. The recruitment strategy aimed to avoid burdening already busy clinicians with the additional task of recruiting to the study.

**Figure 4:**
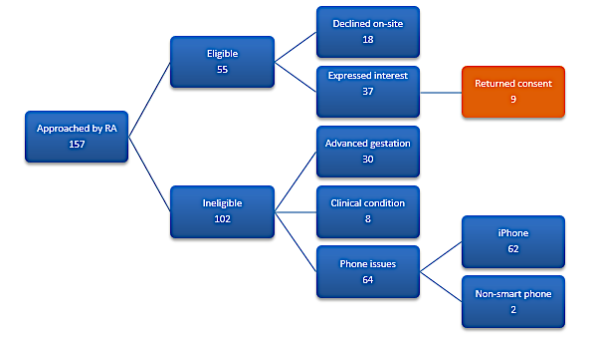
Feasibility study recruitment results. RA: Research Assistant.

Figure 4 illustrates the results of the recruitment. In this period, 157 women were approached and only 9 women were successfully recruited to the study. The majority of women who were ineligible were so because they owned an iPhone rather than Android. Almost 30% were ineligible due to stage of gestation. Over 75% of eligible women who expressed an interest and were provided with a research pack failed to return a signed consent form and baseline questionnaire and thus enroll into the study.

## Results

The project is currently recruiting though with a rate of 100 recruitments in approximately 12 months, recruitment is slower than expected. Additional funds are being sought to enhance recruitment through the employment of additional research assistants and a comprehensive advertising strategy.

## Discussion

### Principal Findings

This protocol aims to determine whether the use of the smartphone app Eating4Two is effective in assisting women to optimize GWG and improve birth outcomes. The intervention takes advantage of smartphone technology, addresses the problem of the limited health service resources available to meet an expanding area of need, and focuses on empowering women to manage their weight in partnership with their maternity care provider. In addition, it addressed the question of whether the intervention impacts differently women with varying levels of relative socioeconomic advantage and disadvantage.

Our feasibility study clearly identified problems in both the recruitment strategy and in the app platform. Although iOS (iPhones) is more popular with younger populations [48], both the Android and iOS providers dominate the field in Australia, and providing the app in both platforms will greatly increase study eligibility.

The recruitment strategy was also revised to involve a range of maternity care service providers including general practitioners, private obstetricians, employed obstetricians, and midwives. Most women visit their general practitioner to confirm their pregnancy, and this makes this group particularly important to ensure that women are recruited early to resolve the other main issue compromising eligibility of advanced gestation. Relying on clinicians can be a risky recruitment strategy as they are often busy and prioritize clinical care. There are, however, a number of strategies that will be employed to enhance the strategy including early engagement; study logo; and regular communication through a study newsletter, eg, acknowledging participation (with merit letters or certificates for example) and showing appreciation [49].

### Digital Preservation

During the trial period, the app will only be available to research participants only, though source coding and App content have been preserved. Researchers interested in replicating this trial will be invited to contact the researchers directly. Health care interventions such as the Eating4Two app must be evaluated carefully to ensure no harm is brought to the pregnant woman or fetus.

### Limitations

The study relies on self-reported height and weight to establish the BMI of participants in the intervention group. Maternity care providers usually calculate BMI at the first antenatal booking visit, and participants can correct these data within the application if necessary. Nonetheless, it is reliant on the accuracy of the participant’s entry. Total weight gain is established by subtracting the woman’s first recorded pregnancy weight from her recorded weight in labor, and we will be unable to vouch for the accuracy of the scales used in various antenatal and labor and birth settings. The quality of the scales used and the procedures for maintaining and calibrating them may vary. Although this may affect the results of the study, it is important to test methods that will be used in translating the intervention to service delivery if successful. Finally, this study does not include any follow-up of women to assess their longer-term weight change post birth.

### Conclusions

Many women gain excessive weight during pregnancy, and this causes problems for the index pregnancy and contributes to the burden of overweight and obesity in society, as excessive gestational weight is often retained postpregnancy. To date, research has focused on intensive interventions that are costly for health services and are unlikely to be sustainable. Few studies have examined the way that women from varying socioeconomic positions might receive interventions. Maternity caregivers are often also reticent about raising the issue of weight with women, even though gestational weight gain is a clinically important issue. The Eating4Two mobile phone app is an intervention that if shown to be effective could be scalable and cost-effectively implemented throughout Australia.

## Abbreviations

ACT: Australian Capital Territory
BMI: body mass index
GWG: gestational weight gain
IOM: Institute of Medicine
SMS: short message service
IRSAD: Index of Relative Socioeconomic Advantage and Disadvantage
